# Long-latency suppression of auditory and somatosensory change-related cortical responses

**DOI:** 10.1371/journal.pone.0199614

**Published:** 2018-06-26

**Authors:** Nobuyuki Takeuchi, Shunsuke Sugiyama, Koji Inui, Kousuke Kanemoto, Makoto Nishihara

**Affiliations:** 1 Neuropsychiatric Department, Aichi Medical University, Nagakute, Japan; 2 Department of Psychiatry and Psychotherapy, Gifu University, Gifu, Japan; 3 Institute of Human Developmental Research, Aichi Human Service Center, Kasugai, Japan; 4 Department of Integrative Physiology, National Institute for Physiological Sciences, Okazaki, Japan; 5 Multidisciplinary Pain Center, Aichi Medical University, Nagakute, Japan; Harvard Medical School, UNITED STATES

## Abstract

Sensory suppression is a mechanism that attenuates selective information. As for long-latency suppression in auditory and somatosensory systems, paired-pulse suppression, observed as 2 identical stimuli spaced by approximately 500 ms, is widely known, though its mechanism remains to be elucidated. In the present study, we investigated the relationship between auditory and somatosensory long-latency suppression of change-related cortical responses using magnetoencephalography. Somatosensory change-related responses were evoked by an abrupt increase in stimulus strength in a train of current-constant square wave pulses at 100 Hz to the left median nerve at the wrist. Furthermore, auditory change-related responses were elicited by an increase in sound pressure by 15 dB in a continuous sound composed of a train of 25-ms pure tones. Binaural stimulation was used in Experiment 1, while monaural stimulation was used in Experiment 2. For both somatosensory and auditory stimuli, the conditioning and test stimuli were identical, and inserted at 2400 and 3000 ms, respectively. The results showed clear suppression of the test response in the bilateral parisylvian region, but not in the postcentral gyrus of the contralateral hemisphere in the somatosensory system. Similarly, the test response in the bilateral supratemporal plane (N100m) was suppressed in the auditory system. Furthermore, there was a significant correlation between suppression of right N100m and right parisylvian activity, suggesting that similar mechanisms are involved in both. Finally, a high test-retest reliability for suppression was seen with both modalities. Suppression revealed in the present study is considered to reflect sensory inhibition ability in individual subjects.

## Introduction

A preceding sensory stimulus attenuates the response to a following stimulus, which is considered to reflect inhibitory processes and sometimes referred to as sensory suppression. Although these suppression mechanisms have yet to be fully elucidated, they have been extensively studied, especially in regard to auditory sense [1]. Such suppression is based on sensory memory [2, 3], thus novel sounds diminish responses to succeeding stimuli [4, 5], while suppression is also observed as other than simple sound changes, such as spatial change [6], and incongruence between auditory and visual information [7]. As for the responsible neural mechanisms, both a pyramidal cell-pyramidal cell depressing synapse and an inhibitory circuit are possible [5, 8].

Paired pulse suppression, an electrophysiological measurement of cortical responses to 2 consecutive identical auditory stimuli spaced 500 ms apart [9, 10], is used to detect long-latency suppression, in which the amplitude of an evoked response at approximately 50 ms (P50) is compared between the first and second stimuli. This measurement technique is clinically important, as previous studies have shown deficits in paired-pulse suppression in patients with schizophrenia [10–14], bipolar disorder [15], panic disorder [16], epilepsy [17], and attention-deficit/hyperactive disorder [18]. In addition, paired-pulse suppression is also related to increased risk for developing schizophrenia [19] as well as deficits in attention processing in schizophrenia [20]. Hence, a paired-pulse suppression paradigm is expected to be useful in a wide range of clinical situations.

As for somatosensory suppression, similar to auditory paired-pulse suppression, responses in the secondary somatosensory cortex contralateral on the stimulated side (cSII) to the second stimulus are suppressed in healthy individuals [21–23]. On the other hand, patients with schizophrenia were found to have deficits in SII suppression, but not in the primary somatosensory cortex (SI) [24], whereas there were no deficits of suppression in those with autism spectrum disorder [25]. In patients with fibromyalgia, somatosensory suppression was shown to be impaired, while auditory paired pulse suppression was normal [26].

Change-related cortical responses are specifically elicited by an abrupt change in a continuous sensory stimulus, and can be clearly recorded using magnetoencephalography (MEG) or electroencephalography (EEG) without subject attention required [27–31]. Because these activities show high test-retest reliability [32–34], the results are considered to be reliable for examining higher order brain functions. Change-related activities are present in the somatosensory, visual, and auditory systems [35–39], and we recently developed methods to observe sensory suppression using change-related cortical responses [5, 34, 40]. As for long-latency suppression of auditory change-related responses, we previously found that suppression peaks at a conditioning-test interval (CTI) of 500–700 ms with modest effects at shorter CTIs, a weak leading stimulus that itself evokes only a weak or no response to cause suppression, and that long-latency suppression has high thresholds as compared to short-latency suppression [5, 41]. Based on those findings, we speculated that suppression reflects long-latency inhibitory postsynaptic potentials (IPSPs) via a specific type of interneuron, with the most probable candidate Martinotti cells, as they induce long-latency IPSPs and have higher thresholds than other cells [42]. It is considered that the purpose of such mechanisms is to prevent runaway of pyramidal cells.

Our paradigm may be useful for evaluating inhibitory function in individual subjects in clinical situations, because many diseases such as epilepsy [17] are considered to cause deficits in inhibitory mechanisms. However, it remains unclear whether inhibition of a specific auditory system reflects the fundamental functions of inhibitory circuits in an individual, as only a few studies have evaluated the relationships among auditory and sensory systems in healthy subjects [43]. In this regard, it is known that the same laminar organization and patterns of connections are present throughout the neocortex [44, 45]. As for basket and Martinotti cells, the major classes of interneurons, non-specific dense connections to neighboring pyramidal cells, which function to blanket inhibition, have been reported in whole-cell recording studies [46, 47]. Furthermore, the anatomical connections of Martinotti cells and their long-latency IPSPs are similar across cortical areas and species [47–49].

In the present study, we utilized MEG to record long-latency suppression using change-related cortical responses, and then examined correlations between the somatosensory and auditory systems. Given that suppression comes from a synaptic pathway ubiquitous across sensory cortices [44], we found a significant association.

## Methods

The study protocol was approved in advance by the Ethics Committee of the National Institute for Physiological Sciences, Okazaki, Japan, and all subjects provided written consent prior to participation. None had a history of mental or neurological disorders, or substance abuse in the most recent 5 years, and all were free of medication at the time of testing.

### Auditory stimuli

**Fig 1:**
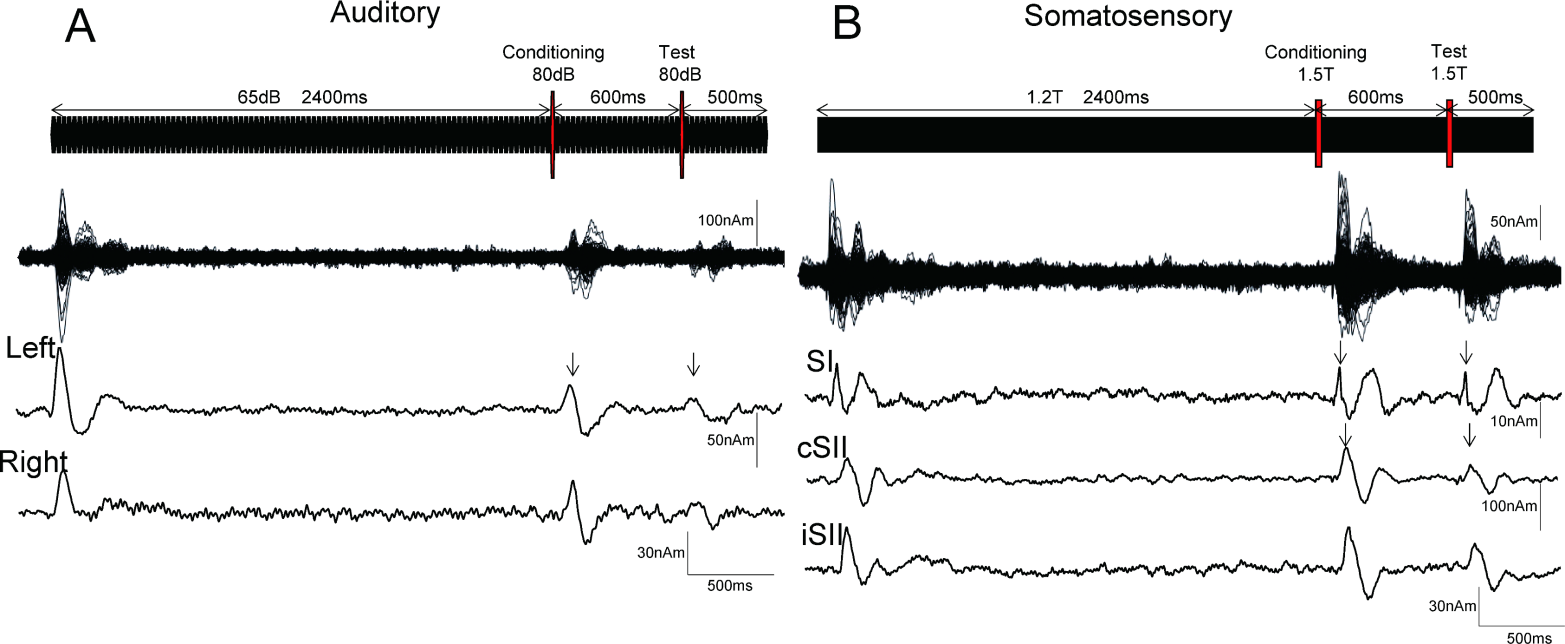
Paired stimulation paradigm using auditory and somatosensory change-related cortical responses. Data from a representative subject are presented. Shown is the stimulation paradigm, superimposed MEG waveforms from all 204 sensors, and source strength waveforms for each cortical activity in the auditory (A) and somatosensory (B) experiments. T, sensory threshold. Arrowheads show peaks of source activity used for amplitude measurements.

Repeats of a 25-ms pure tone at a frequency of 800 Hz were used (rise/fall, 5 ms). The sound was created by 140 repeats of a 25-ms tone at 65 dB SPL of sound pressure, yielding a total duration of 3500 ms. Fig 1 shows the stimulation paradigm. For the test sound, a 25-ms tone at 80 dB was inserted at 3000 ms. The conditioning stimulus was also 25 ms in duration and 80 dB in sound pressure, and presented at 600 ms before the test stimulus. Thus, the CTI was 600 ms. In Experiment 1, sound stimuli were presented binaurally, while in Experiment 2 they were presented only to the left side. Ear pieces (E-A-Rtone 3A, Aero Company, Indianapolis, IN) were used in each experiment. Pure tones instead of clicks were used in the present study based on a previously reported auditory long-latency suppression paradigm showing conditioning stimuli to be a substitute for a Test alone response [41] and because results of our preliminary study showed that pure tones could elicit clearer change-related cortical responses as compared to clicks. It has also been shown that change-related cortical responses are elicited by an abrupt change in sound feature, regardless of whether the sound is composed of clicks or pure tones [28].

### Somatosensory stimuli

A train of current-constant square wave pulses (pulse duration, 0.5 ms) at 100 Hz was applied to the left median nerve at the wrist using a felt-tip bipolar electrode for 3500 ms. For the test and conditioning stimuli, 2 pulses at 100 Hz were inserted at 2400 and 3000 ms, respectively, so as to be similar to the auditory stimuli. The intensity of the test and conditioning stimuli was 1.5 times that of the sensory threshold, and that of the background pulse was 1.2 times above the threshold. We used a CTI of 600 ms for both auditory and somatosensory suppression. It has been shown that auditory suppression has at least 3 peaks at CTIs of 10–30, 40–60, and 500–700 ms [5, 41]. As for somatosensory long-latency suppression, some studies have tested effects of a CTI in which SI was suppressed at least until 100–200 ms and SII until 500–800 ms [21, 23, 50].

### Recordings

Each subject sat in a chair and watched a silent movie on a screen placed 2 m in front of them, and was instructed to ignore sound and somatosensory stimuli throughout the experiment. Magnetic signals were recorded using a 306-channel whole-head type MEG system (Vector-view, ELEKTA Neuromag, Helsinki, Finland), which was comprised of 102 identical triple sensor elements. Each sensor element consisted of 2 orthogonal planar gradiometers and 1 magnetometer coupled to a multi-superconducting quantum interference device (SQUID), thus providing 3 independent measurements of the magnetic fields. In the present study, we analyzed MEG signals recorded from 204 planar-type gradiometers, which were sufficiently powerful to detect the largest signal just over local cerebral sources. Signals were recorded with a bandpass filter of 0.1–300 Hz and digitized at 1000 Hz. Auditory and somatosensory stimuli were randomly presented. Analysis was conducted from 100 ms before to 4000 ms after stimulus onset. The period of 2300–2399 ms was used as the DC offset. Epochs with MEG signals larger than 2.7 pT/cm were rejected from averaging.

### Procedures

#### Experiment 1

We examined the correlation between somatosensory suppression following left median nerve stimulation and auditory suppression following binaural stimulation in 13 healthy volunteers (10 males, 3 females; mean age 33.0±9.7 years). In previous studies conducted by our group, binaural stimulation has been used for investigating auditory suppression. For the present investigation, somatosensory and auditory stimuli were randomly presented, with at least 100 artifact-free epochs averaged for each somatosensory and sound stimulus.

#### Experiment 2

Eleven subjects from Experiment 1 also participated in Experiment 2 (8 males, 3 females; mean age 34.6±9.7 years), as 2 could not join for personal reasons. The 2 experiments were spaced by more than 2 weeks. The protocol for Experiment 2 was identical to that of Experiment 1, except that auditory stimulation was applied to the left ear alone, with at least 100 artifact-free epochs averaged for each somatosensory and sound stimulus.

### Analysis

Dipole analyses of the responses to the conditioning stimulus were performed using the Brain Electrical Source Analysis (BESA) software package (NeuroScan, Mclean, VA), as previously described [51]. A pass-band filter of 1–100 Hz was used for both somatosensory and auditory responses. The abrupt increase in intensity of the somatosensory stimuli elicited clear magnetic responses in 3 areas; the parietal area contralateral to the stimulation and temporal area in both hemispheres. We measured the peak latency and amplitude of the parietal component peaking at 40–100 ms, contralateral temporal component at 40–110 ms, and ipsilateral temporal component at 70–160 ms [31]. The percent inhibition of the test response by the conditioning stimulus (%suppression) was calculated as follows: (Conditioning response–(Conditioning + Test response) / Conditioning response)*100 [41]. We then compared the values for %suppression between the somatosensory and auditory systems within each experiment, and between both experiments.

## Results

### Experiment 1

**Fig 2:**
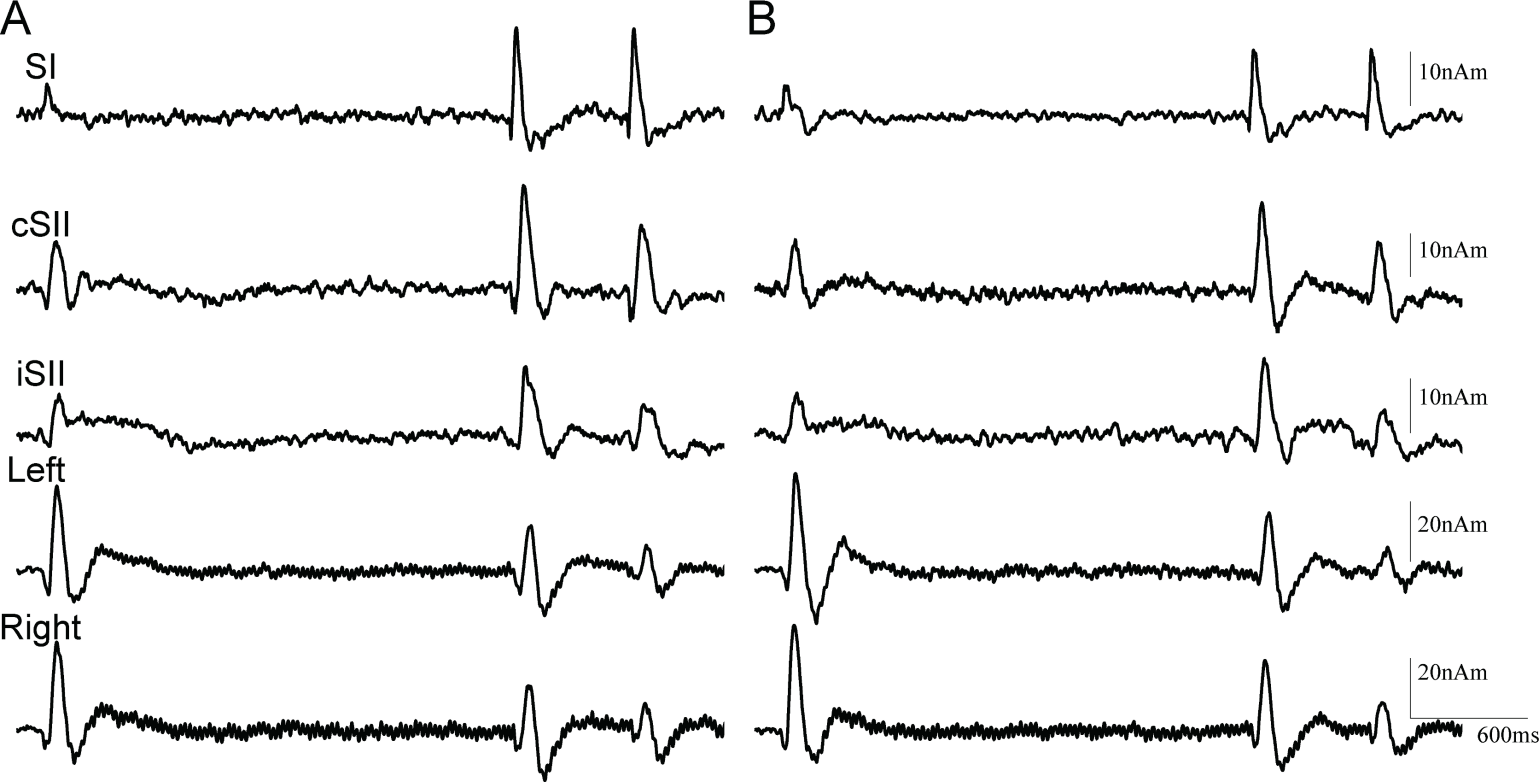
Grand-averaged waveforms for all subjects. Test responses, except for SI, were suppressed in both Experiment 1 (A) and 2 (B).

**Fig 3:**
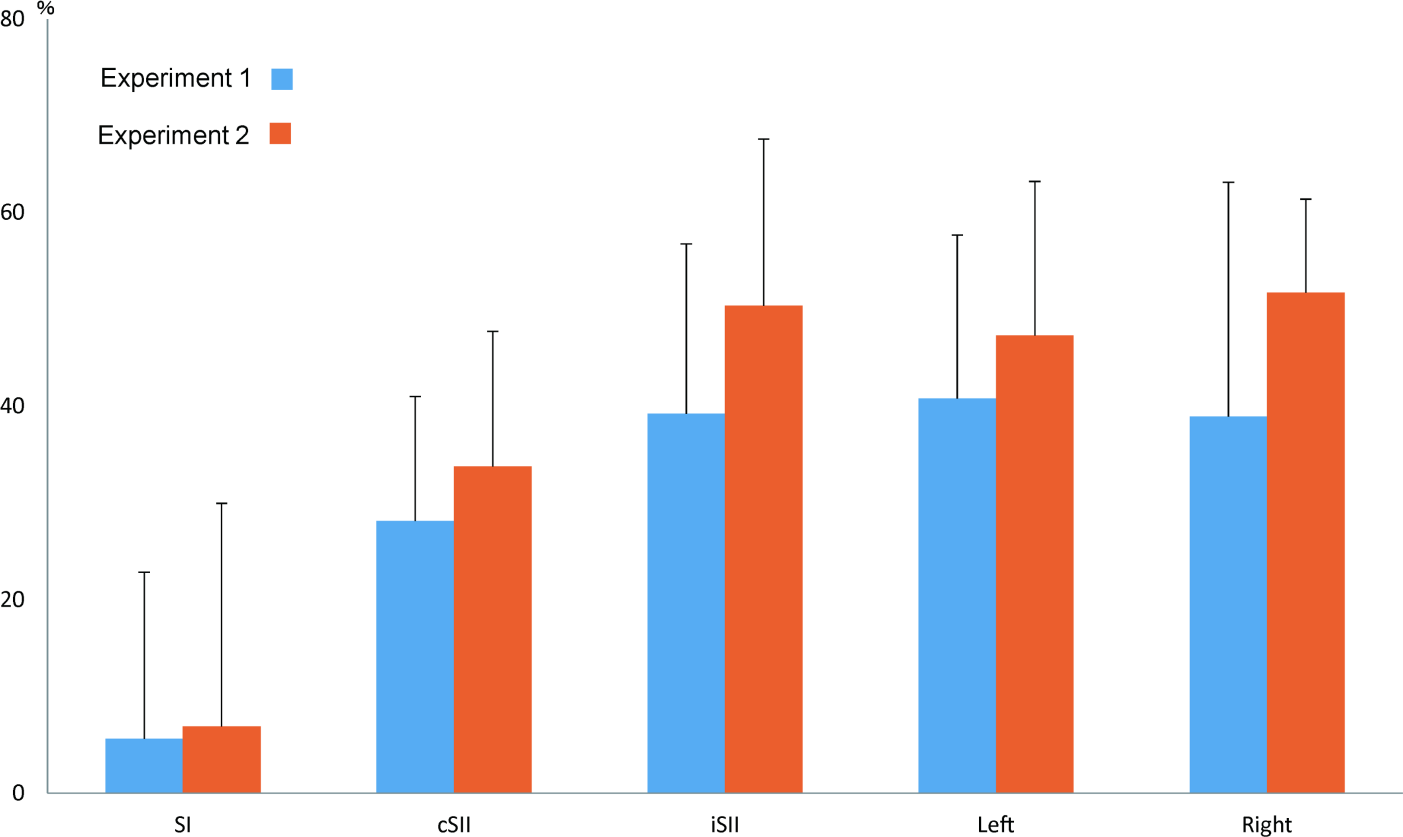
Mean %suppression value for each cortical activity. Values are shown as the mean ± SD. All cortical activities, except for SI, showed a significant reduction in amplitude for the test response.

Somatosensory change-related responses were evoked in 3 cortical areas. Dipoles were estimated to be located in the postcentral gyrus of the contralateral hemisphere (SI) and contralateral perisylvian region including SII. All subjects had activity in SI, while cSII and iSII were activated in 12 of 13. Fig 1 shows the original MEG and source strength waveforms of a representative subject, with grand-averaged waveforms for each activity in both experiments shown in Fig 2. Table 1 and Fig 3 show %suppression in both Experiment 1 and 2. Response to the test stimulus (test response) was significantly smaller in amplitude as compared to the response to the conditioning stimulus (conditioning response) for cSII (p = 0.012) and iSII (p = 0.003), but not for SI (p = 0.52). There was no correlation between cSII and iSII for %suppression (r^2^ = 0.09, p = 0.41).

**Table 1:** Amplitude and rate of inhibition in Experiment 1 and 2.

	Amplitude (nAm)						%suppression (SD)		
	Conditioning (SD)			Test (SD)					
	SI	cSII	iSII	SI	cSII	iSII	SI	cSII	iSII
Experiment 1	18.9 (10.8)	27.8 (19.7)	17.5 (11.0)	18.1 (11.7)	19.4 (12.0)	10.1 (6.4)	5.62 (5.8)	28.1 (27.7)	39.2 (37.1)
Experiment 2	16.1 (6.9)	24.1 (13.4)	18.5 (7.8)	15.5 (8.5)	15.5 (7.9)	9.04 (5.0)	6.90 (23.0)	33.7 (14.0)	50.4 (17.2)
	L		R	L		R	L		R
Experiment 1	19.2 (10.0)		20.5 (9.2)	10.9 (4.1)		11.4 (6.0)	40.8 (40.9)		38.9 (38.3)
Experiment 2	22.4 (14.7)		27.2 (10.8)	10.9 (6.4)		13.1 (5.6)	47.3 (15.9)		51.7 (9.7)

The equivalent current dipole for the main component of the auditory evoked responses, N100m, was estimated in and around the supratemporal plane of both hemispheres [41] in all subjects. Similar to SII, N100m was suppressed by the conditioning stimulus (Fig 1B) in both the left (p = 2.8*10^−3^) and right (p = 8.7*10^−3^) hemispheres, and the degree of suppression was correlated between the hemispheres (r^2^ = 0.63, p = 1.2*10^−3^).

When %suppression was compared between the somatosensory and auditory systems, the correlation coefficient r^2^ value between cSII and right N100m was 0.27 (p = 0.08), while that between iSII and left N100m was 0.35 (p = 0.06). There was no correlation between SI and right N100m (correlation coefficient, r^2^ = 0.02, p = 0.65). There was also no significant difference in latency between the conditioning and test responses for SI (p = 0.73), cSII (p = 0.59), iSII (p = 0.96), left N100m (p = 0.31), and right N100m (p = 0.14). The peak latency values for each cortical activity are shown in Table 2.

**Table 2:** Latency in Experiment 1 and 2.

	Latency (ms)					
	Conditioning (SD)			Test (SD)		
	SI	cSII	iSII	SI	cSII	iSII
Experiment 1	56.8 (14.8)	87.8 (9.1)	118 (30.2)	55.5 (19.3)	85.5 (21.1)	119 (30.3)
Experiment 2	58.5 (17.4)	86.2 (12.9)	119 (32.2)	58.1 (17.7)	86.4 (18.3)	120 (30.5)
	L		R	L		R
Experiment 1	120 (9.6)		115 (11.4)	117 (10.5)		108 (11.7)
Experiment 2	119 (10.7)		104 (10.2)	117 (20.5)		100 (16.3)

### Experiment 2

All subjects showed activity in SI, cSII, and auditory N100m, and 10 of 11 had activity in iSII. The effects of the conditioning stimulus were similar to those in Experiment 1, with significant suppression of the test response for cSII (p = 0.012), iSII (p = 2.6*10^−3^), left N100m (p = 3.1*10^−3^), and right N100m (p = 2.9*10^−5^), but not for SI (p = 0.54). Furthermore, %suppression was correlated between cSII and right N100m (r^2^ = 0.57, p = 0.008), but not between iSII and left N100m (r^2^ = 0.06, p = 0.50). Also similar to the results of Experiment 1, the degree of suppression was correlated between left and right N100m (r^2^ = 0.36, p = 0.052). Peak latency did not differ significantly between the conditioning and test responses for both the somatosensory and auditory systems (p >0.36).

### Correlations between Experiment 1 and 2

**Fig 4:**
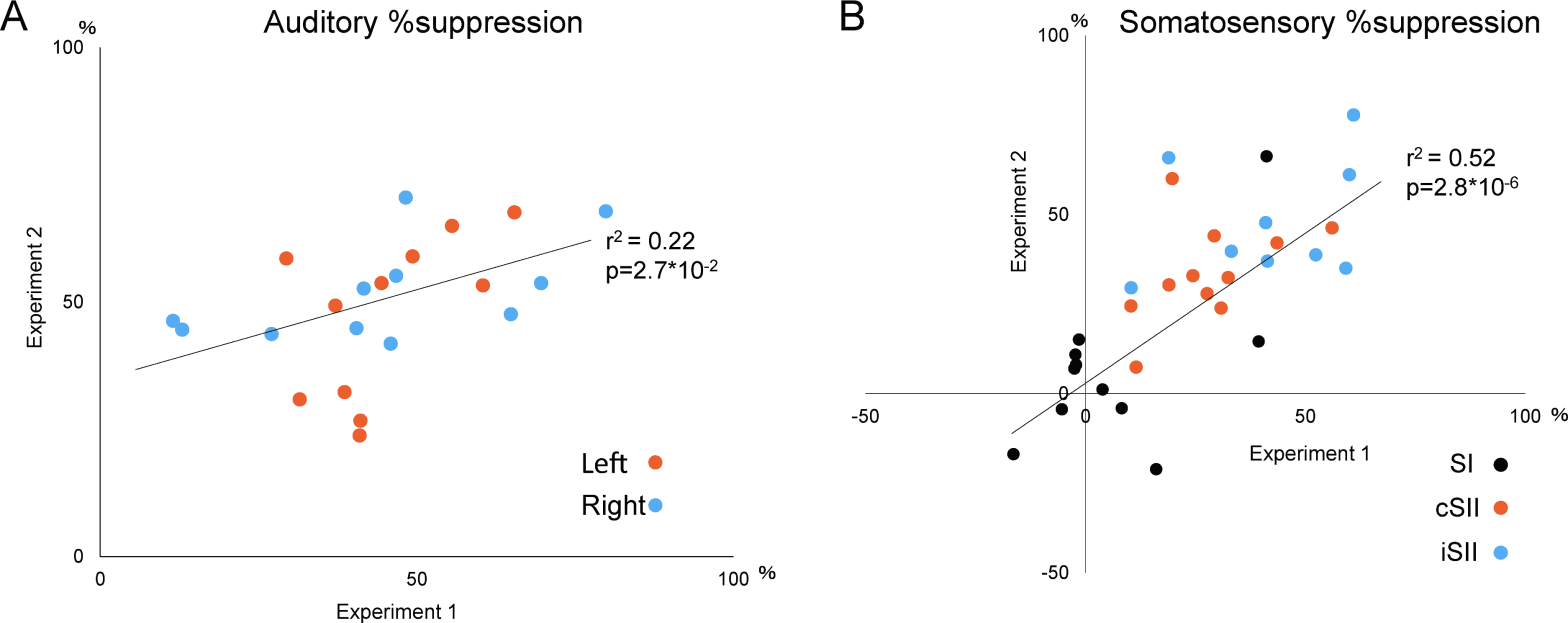
Correlation of %suppression between experiments. Plots showing the relationship of %suppression between Experiment 1 (x axis) and Experiment 2 (y axis) for the auditory (A) and somatosensory (B) experiments. The r and p values presented were obtained from all collected data.

**Fig 5:**
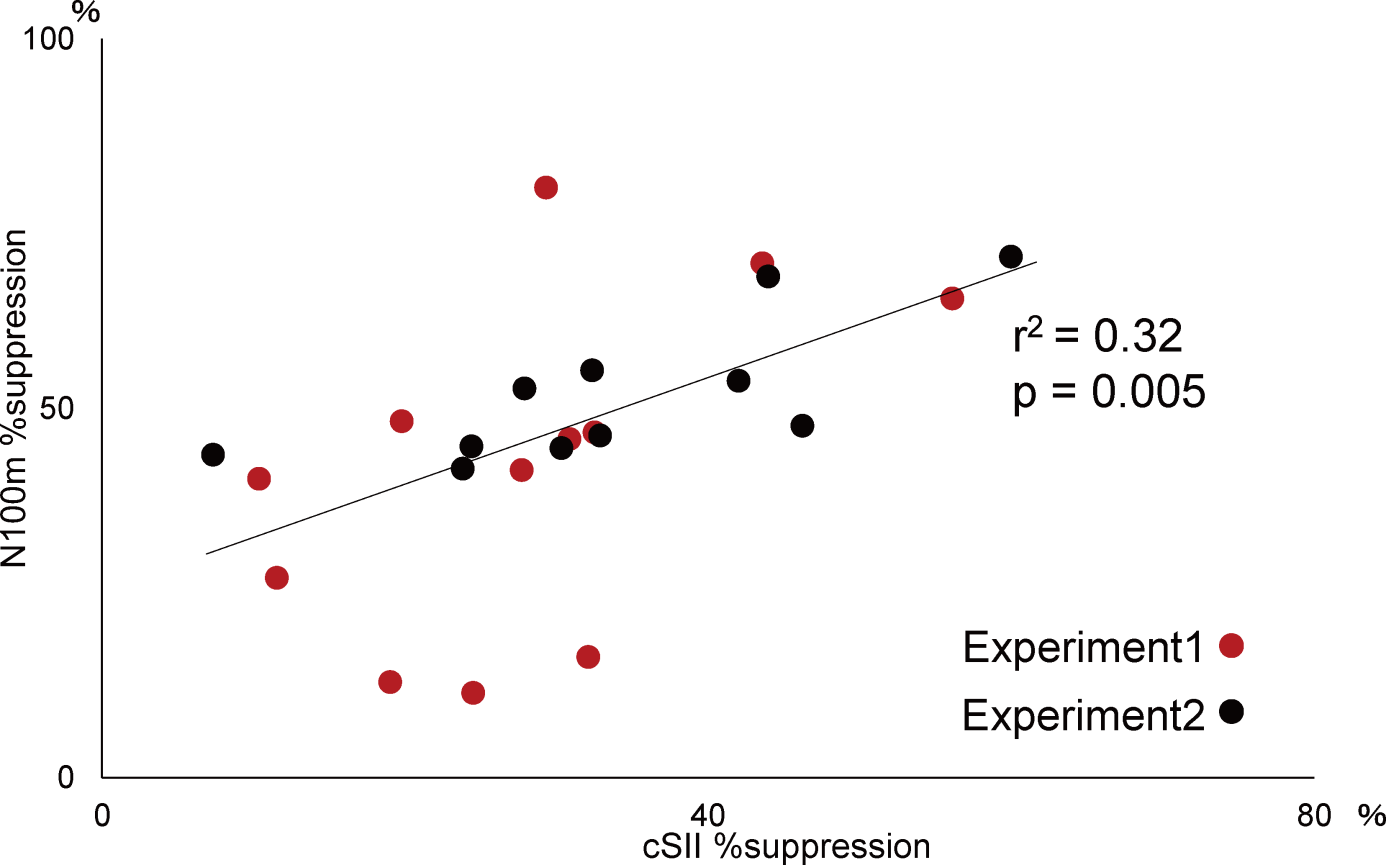
Correlation of %suppression between cSII and right N100m. Plots showing the relationship of %suppression between cSII (x axis) and N100m (y axis).

Fig 4 shows %suppression values in Experiment 1 and 2. There was a significant correlation between the experiments for N100m (r^2^ = 0.22, p = 0.027) (Fig 4A) and somatosensory responses (r^2^ = 0.52, p = 2.8*10^−6^) (Fig 4B). When data obtained in both experiments were compared, a significant correlation was seen between cSII and right N100m (r^2^ = 0.32, p = 0.005) (Fig 5).

## Discussion

### Relationship between tactile and auditory long latency suppression

Auditory suppression was significantly correlated between the 2 present experiments despite different settings, in which the sound was presented in a binaural manner in Experiment 1 and monaurally in Experiment 2. Similar to SII, the most lateral part of Heschl’s gyrus or the superior temporal gyrus, which is considered to be the secondary auditory cortex (AII) [51], is bilaterally activated by sensory inputs or abrupt changes in a continuous sensory stimulus. Behaviors of SII and auditory STG activities are similar in some aspects, including sensitivity to inter-stimulus intervals for onset response [38], sensitivity to change in magnitudes for change-related response [28, 35], and suppression mechanisms [43]. Therefore, we consider that these 2 cortical areas have similar physiological functions including change detection. In the present experiments, the degree of suppression was correlated between N100m and SII, suggesting the existence of a similar inhibitory mechanism between auditory and somatosensory change-detecting systems.

In this study, we did not record responses in the primary auditory cortex (AI), because of its deep location and MEG is only able to detect signals weakly from deep brain areas. On the other hand, magnetometers can separate activities from primary and secondary auditory areas [51]. In future studies, it will be necessary to compare activities in different auditory areas. Jääskeläinen et al. showed differences in regard to adaptation across different parts of the auditory cortex [4]. The properties of S2 suppression by preceding stimuli resemble those previously documented for non-primary auditory cortex areas.

The principal organization of the neocortex in cortical areas is relatively uniform [44, 52] and inhibitory innervation of neighboring pyramidal cells, a basic structure of the cerebral cortex, is similar [53]. Furthermore, the same basic brain structure throughout the neocortex is applicable to inhibitory neurons [45]. As for Martinotti cells, they are densely connected to nearby pyramidal cells and inhibit them in a non-specific manner throughout the cerebral cortex [46, 54]. Since the same basic structure exists across the sensory cortices and the same cells are involved in suppression, a similar degree of suppression across sensory modalities is expected in individual subjects. The present results and EEG data [43] support this speculation, as we found a correlation in regard to suppression between the somatosensory and auditory systems, as well as between left and right N100m. Furthermore, they indicate the possibility that long-latency suppression of any sensory modality reflects the ubiquitous inhibitory mechanism of an individual. To confirm this, additional empirical data are necessary, such as suppression in visual and pain systems.

### Somatosensory suppression

We noted clear suppression of the test response seen in cSII and iSII, but not in SI, results that are consistent with previous studies that reported SII suppression with a conditioning stimulus presented at 500 ms before the test stimulus [23, 55]. In the present study, the degree of suppression was not correlated between SI and SII, and %suppression for SI was very weak in spite of an approximately 30% suppression of cSII activity. In addition, findings of previous anatomical [56, 57] and electrophysiological [50, 58, 59] studies support the presence of serial and hierarchical processing through SI and SII, suggesting greater or specific inhibitory mechanisms for SII.

There are 2 possibilities for the origin of iSII activation; cSII via the corpus callosum [60, 61] and sequential activation in the ipsilateral hemisphere driven by direct inputs from the ipsilateral periphery [60, 62]. In our study, we found no significant relationship between iSII and cSII in regard to inhibition rate, suggesting that suppression of iSII activity is not dependent on cSII suppression, thus indicating that iSII and cSII receive their own inhibitory inputs. However, we also noted that iSII had a lower signal-to-noise ratio than cSII, which might contribute to mask their correlation.

There was no significant suppression in SI, though it is possible that we could not detect SI suppression because of the low signal to noise ratio or methodological problems. In neuroimaging and cellular level studies, when long latency suppression was lacking, SI suppression was indicated [63] and SI suppression is known to have a relationship with chronic pain [64]. Electrophysiological studies have shown several types of suppression in relation to an effective conditioning-test interval [5, 8]. Additional studies are necessary to clarify the suppression mechanisms in the primary sensory cortex.

### Somatosensory suppression paradigm

In the present study, somatosensory suppression showed a high level of test-retest reliability (r^2^ = 0.52, Fig 4), supporting the possibility of its usefulness as a clinical test. Furthermore, our results showed suppression in SII of 30–50%, though only 10% in SI. Therefore, it is possible that each component shows specific changes under certain clinical conditions. This information may also be useful for testing conducted with EEG.

We did not use a test alone condition in the present examinations, because responses to the conditioning stimulus do not differ from those to the test stimulus when there is an adequate steady state duration prior to the conditioning stimulus [41]. Similar to auditory change-related cortical responses, it is known that change-related somatosensory responses are dependent on past sensory history. When the duration of the steady state prior to the change onset is varied, the amplitude of the change-related response increases steeply at 500–1500 ms, whereas the increment becomes more modest at longer durations because of the non-linear temporal nature of haptic memory [35]. Therefore, for the present examinations the duration of the steady state prior to the test stimulus was set at 2400 ms.

Approximately 6 minutes was required for the present somatosensory paradigm. Somatosensory suppression is an important sensory mechanism that requires further elucidation, though it is expected to find use in clinical situations [24–26]. Particularly, patients with autism or fibromyalgia may have deficits specifically in the somatosensory inhibitory mechanism [25, 26]. Taken together, the present paradigm is anticipated to become a useful clinical tool.

## Conclusions

In the present study, we examined paired pulse suppression using change-related cortical activity, and compared the degree of suppression between the auditory and somatosensory systems. Our results showed a significant correlation between those systems as well as between the hemispheres for the auditory system. In addition, they suggest that the present measurement findings reflect the effects of long-latency inhibitory circuits in individuals. A variety of diseases are known to be related to deficits in sensory inhibition, thus such a functional measurement method may be useful for assessment of the inhibitory system.
